# Benefits, barriers and facilitators for out-of-hospital point of care testing: a qualitative study

**DOI:** 10.3310/nihropenres.13583.1

**Published:** 2024-06-27

**Authors:** Jessica Coggins, Kim Kirby, Richard Body, Jonathan Benger

**Affiliations:** 1 School for Health and Social Wellbeing, University of the West of England, Bristol, England, BS16 1DD, UK; 2 South Western Ambulance Service NHS Foundation Trust, Exeter, EX2 7HY, UK; 3 The University of Manchester, Manchester, England, M13 9PL, UK; 4 Emergency Department, Manchester University NHS Foundation Trust, Manchester, M13 9WL, UK

**Keywords:** Point of care systems, ambulances, safety

## Abstract

**Background:**

Heightened pressures on hospitals and Emergency Medical Services (EMS) due to growing demand and staff shortages have led to prolonged ambulance response times and delays in handing over the care of EMS patients on arrival at an Emergency Department. These delays jeopardise patient safety and staff wellbeing. Point of care (POC) tests in EMS have been proposed to facilitate effective on-scene decision-making, reduced conveyance, improved clinical outcomes, enhanced system efficiency and patient experience. Despite an acceleration in POC testing during the Covid-19 pandemic, limited evidence exists for integrating POC tests into routine EMS practice. The aim of this research was to explore the impact, benefits, barriers, and facilitators of POC testing in United Kingdom (UK) EMS, alongside factors influencing future research on POC testing implementation.

**Methods:**

Convenience and snowballing sample techniques were used to recruit a diverse stakeholder group, including patient and public participants, for online semi-structured interviews between June and July 2023. Interviews were recorded, transcribed verbatim and thematically analysed using the framework method. The codes were pre-selected using the outcomes of a prior stakeholder event and double coded by the research team.

**Results:**

A total of 25 participants took part in semi-structured interviews. Whilst most participants identified clear potential benefits associated with the implementation of further POC tests within UK EMS, barriers that need to be considered in future research were also highlighted. Three themes were identified: enhancing patient care and system efficiency through POC testing; navigating implementation challenges: balancing barriers and facilitators for successful implementation; building the foundations: key considerations for future research.

**Conclusions:**

Our study indicates that although the adoption of further POC testing was viewed positively, with substantial potential for impact, it will be essential to carefully address the barriers identified, along with insights from prior research, to overcome the associated challenges effectively.

## Plain Language Summary

Ambulance services in the United Kingdom (UK) are under significant strain due to increasing demand and staff shortages. This results in longer ambulance response times and delays in handing over patient care at emergency departments. These delays have a negative impact on patient safety and the wellbeing of staff. Portable medical tests carried out where the patient is being treated are known as “point of care” tests. Advances in technology mean that these tests are now increasingly available. Their use in ambulance services could help with decision-making, reducing the need for patients to be transported to a hospital, improving patient outcomes, enhancing patient experience, and improving system efficiency.

However, research on point of care testing in ambulance services is limited. Therefore, this study aimed to explore the perceived impact, benefits, barriers, and facilitators of point of care testing in UK ambulance services, as well as the factors likely to influence future research.

Twenty-five participants, including various healthcare professionals and members of the public, were interviewed using questions developed by the research team. Three main themes emerged from the interviews:

1. Ways in which point of care testing could potentially enhance patient care and system efficiency.
2. A description and exploration of the different barriers and facilitators associated with the implementation of point of care testing.
3. Key considerations for future research on point of care testing.

The study concluded that while the adoption of additional point of care testing within ambulance services was viewed positively and has the potential for significant impact, it will be crucial to address the identified considerations and barriers carefully if successful outcomes are to be achieved.

## Introduction

In the United Kingdom (UK) National Health Service (NHS) and many other countries, hospitals and Emergency Medical Services (EMS) are experiencing heightened pressures due to growing demand and staff shortages. These pressures have resulted in prolonged ambulance response times and delays in handing over the care of EMS patients on arrival at an Emergency Department (ED, ^1^). Delays in EMS handover raise concerns regarding patient safety, both for patients waiting to be admitted to an ED and for those waiting for an ambulance ( 2; www.cqc.org.uk/ambulance-handovers). A detrimental impact on staff wellbeing has also been highlighted ( www.cqc.org.uk/ambulance-handovers).

In response to these increased pressures NHS England has published a programme that aims to safely reduce avoidable EMS conveyance by supporting skilled paramedics to treat patients at home or in a more appropriate setting outside of the ED, with the aim of reducing ED crowding and avoiding unnecessary inter-hospital transfers ( https://www.england.nhs.uk/National-guidance-for-ambulance-clinician-referral-to-same-day-emergency-care.pdf; https://www.england.nhs.uk/planning-to-safetly-reduce-avoidable-conveyance-v4.0.pdf). Within this programme there is a focus on evaluating new opportunities for safely reducing conveyance. 

In practice, the conveyance decision-making process is often complex and multifactorial, and there is a limited number of tools available to support these decisions ^3^. Point of care (POC) tests have been proposed as a practical means of supporting on-scene decision-making, with potential to reduce conveyance to the ED, enable the provision of correct treatments at an earlier stage, enhance healthcare system efficiency, and ultimately benefit patient outcomes and experience ^4, 5^. POC testing adoption accelerated during the recent Covid-19 pandemic ^6, 7^ but there is limited evidence to support integrating further POC tests into routine practices, particularly in EMS and out-of-hospital care. Furthermore, POC tests which appear to meet clinical requirements may fail to be successfully implemented due to the value proposition not being understood by decision makers ^8^.

Before further POC tests are considered for implementation in UK EMS, it is essential to explore the factors that will affect successful adoption. Therefore, a qualitative interview study was designed which aimed to explore the impact, benefits, barriers, and facilitators of POC testing within UK EMS. It also aimed to explore the factors that could either support or hinder future research on POC implementation.

## Methods

### Patient and public involvement

The study benefited from the input of several public contributors throughout the design and delivery of the wider study. The public contributors helped the research team understand their experiences with varied service provision, provided their perspectives on pre-hospital testing, and subsequently informed the topic guides for the interviews ^9^. Additionally, the public contributors reviewed the results following data analysis.

### Study design

This was a qualitative semi-structured interview study, underpinned by an exploratory-descriptive qualitative approach ^10^.

### Recruitment and consent

A diverse stakeholder group, who met the eligibility criteria in Table 1, were established to support the study. Stakeholders were identified and recruited through the existing professional networks of the research team. A combination of convenience and snowball sampling was used to recruit up to 20 participants with diverse backgrounds and experiences. A pragmatic approach was adopted, and a sample size of 20 was determined to be sufficiently large to allow a rich understanding and generate new knowledge in this area.

**Table 1.** Eligibility criteria.

Clinicians with expertise in EMS care and pre-hospital diagnostic testing
Researchers with a track record in POC evaluation
Patient and public representatives, with a particular emphasis on inclusion and ensuring effective input from seldom-heard groups
EMS service provider organisations
Commissioners of EMS services
Individuals with expertise in healthcare policy, service delivery and workforce
International experts in EMS and POC testing to provide a wider perspective

Potential participants were contacted by members of the research team by email with details of the study and an invitation to express an interest in participation. Where an interest in participation was indicated they were sent a participant information sheet, privacy notice, consent form and confidentiality agreement and were invited to take part in an online interview via Microsoft Teams. Participants had the opportunity to contact the study team to ask any questions before completing and returning the consent form. Patient and public participants were offered renumeration for their time via vouchers to the value of £25. Professional participants were offered renumeration for their time to their employing organisation.

### Data collection

Semi-structured interviews were conducted by KK, a senior research fellow in paramedic science, using a topic guide developed by the research team and reviewed by public contributors ^9^. The research aims were explained at the beginning of the interview. Interview questions explored use cases for POC tests, potential tests, benefits, facilitators, and barriers. The interviews took place via Microsoft Teams where the data was captured using a recording function with the participant’s permission. The recordings were transcribed and anonymised by an approved supplier with a data processing agreement in place.

### Data analysis

Thematic analysis was employed using the framework method as the objective of the study was clear and known in advance ^11, 12^. Transcripts were imported into Nvivo 14 software and an analytical framework was developed by two members of the research team (KK and JC) after the joint coding of three transcripts informing the creation of a framework method matrix for charting.

A combination of deductive and inductive coding was used. A stakeholder event held prior to this study had already developed the criteria for assessment of candidate POC tests, mapped the clinical pathways associated with the most promising tests and explored facilitators and barriers to the implementation of POC testing. This informed the deductive coding, and the initial codes can be found in Table 2. Following initial joint coding, JC completed the remainder of the analysis.

**Table 2.** Initial codes.

POC testing benefits	Facilitators	Barriers	Potential tests
Potential to change patient pathway	Clear purpose	Shared records	
	Coordination across the patient pathway	Consideration risks	
	Reliability of tests	Resource impact	
	Decision Support		

Ethics committee approval was received from the University of the West of England, Bristol, Faculty Research Ethics Committee on 2 ^nd^ June 2023 (UWE FREC Ref: HAS.23.04.101).

## Results

A total of 25 participants took part in an interview during June and July 2023. The mean interview duration 34 minutes, with a range of 21 minutes to 49 minutes. Table 3 shows the participants’ areas of expertise and country of employment/residence.

**Table 3.** Participant area of expertise and country of employment/residence.

Participant ID	Area of expertise	Country
1	Prehospital care, point of care testing, prehospital research	UK
2	Point of care testing in healthcare	UK
3	Point of care testing in healthcare	UK
4	Point of care testing in healthcare	UK
5	Prehospital care, Prehospital research	UK
6	Point of care testing in healthcare	UK
7	Laboratory medicine, point of care testing in healthcare	Norway
8	Point of care testing in healthcare	UK
9	Prehospital care	UK
10	Prehospital care	UK
11	Prehospital care, point of care testing healthcare	UK
12	Prehospital care	UK
13	Prehospital care, point of care testing in healthcare	Netherlands
14	Prehospital care, point of care testing in healthcare	United States
15	Prehospital care, point of care testing healthcare	UK
16	Point of care testing in healthcare	UK
17	Research methodology relating to diagnostic tests	UK
18	Prehospital care	UK
19	Patient/public contributor	UK
20	Patient/public contributor	UK
21	Patient/public contributor	UK
22	Patient/public contributor	UK
23	Prehospital care, prehospital research, point of care testing healthcare	UK
24	Prehospital care	UK
25	Prehospital care, point of care testing healthcare	UK

Following the framework method three overarching themes were identified:

1. Enhancing patient care and system efficiency through POC testing
2. Navigating implementation challenges: balancing barriers and facilitators for successful implementation
3. Building the foundations: key considerations for future research

### Enhancing patient care and system efficiency through POC testing

***Potential to change patient pathway and achieve optimal treatment outcomes***

All participants acknowledged that there was potential to alter the patient pathway, ensuring patients can be supported appropriately in the right place, first time. POC testing has the capacity to assist in ruling out conditions which would otherwise require ED care, thereby reducing avoidable ambulance conveyance and ED attendances. Additionally, it could ensure the safe management of patients within their homes. This aspect held particular significance for certain demographic groups, including the elderly and residents of nursing homes, for whom transportation could be distressing.

The outcomes of POC testing also had the potential to guide decisions in redirecting patients towards alternative care pathways, such as urgent care facilities, or accelerating the care pathway towards specialised centres such as those providing coronary intervention, rather than a more generic ED. However, pathway variation, often depending on the geographical location of the patient and local service provision, was felt to undermine the realisation of these potential benefits in practice.

*‘This is the missing link which will take us into the new generation of ambulance services which is identifying the care need, try and meet that care need if you can on scene, and if not refer into a service which can meet that care need, the right service.’ – participant 1*

Participants highlighted that the introduction of POC tests might not always alter the patient pathway, resulting in limited impact. For example, participants predicted challenges in gauging when a paramedic should perform a sepsis POC test based on the patient’s presentation; if a paramedic was concerned enough to conduct a sepsis POC test, how would a decision then be reached not to convey the patient based on a negative result. In a scenario involving major trauma, paramedics are juggling various critical factors, and conducting a POC test would likely be a secondary consideration.

*‘If you are worried about sepsis, I find it hard to see how you would do a point of care test and potentially then not transfer somebody to hospital’ – participant 4*
*‘I think if someone in front of you is a sick trauma, they look like a sick trauma you don’t need a lactate to tell you that, the same as sepsis’ – participant 8*

Alongside a change in pathway, POC tests may lead to more optimal treatment outcomes for patients by enabling earlier diagnoses, expediting care, and potentially hastening recovery, particularly in cases like sepsis where rapid deterioration can occur. Furthermore, they could reduce the risk of missing conditions such as myocardial infarction and sepsis, even when patients appear relatively well. The outcomes of POC testing could also enhance the quality of care by providing guidance for specific treatments, such as antibiotics.

*‘The right treatment the right time, the right place and more quickly. That’s just a summary of it. That is the potential benefit.’ – participant 21*

***Decision support***

Specific POC tests have the potential to impact the confidence of paramedics and support decision-making. Participants generally felt that if paramedics were provided with a tool, such as POC testing, that supports decision making and safe non-conveyance they would use it assuming that it is practical. Nonetheless, it was highlighted that a POC test serves as an additional tool and would not replace clinical decision making and judgment.

*‘I think this would give us more autonomy, it would give us more clinical decision-making skills.’ – participant 12*

***Resource availability and savings***

Participants highlighted the financial benefits of POC testing, including reduced conveyance and ED attendance, however, patient safety was considered paramount.

*‘I would imagine more cost-effective pathway wise in the long-term to save those unnecessary admissions.’ – participant 21*

The perception was that implementing POC testing to decrease conveyance rates could increase the availability of ambulances, leading to reduced wait times and alleviating congestion in EDs. Increased fleet availability would subsequently allow ambulances to attend to other patients more promptly, thereby enhancing patient safety. Non-conveyance would also reduce the number of ED and hospital beds required. Nonetheless, the challenge of hospital discharges would continue to persist.

*‘Reduced delays at the emergency department which again increases patient safety in a different way, if you haven’t got so many delays you can get to the other undifferentiated patients into the community quicker.’ – participant 12*

***Reducing stress or inconvenience for patients and families***

Implementing POC testing out of hospital could potentially alleviate the emotional strain associated with ambulance conveyance and ED attendance, with greater comfort for both patients and their family members, particularly for those who are less mobile. If non-conveyance is the outcome, this would avoid patients having to wait in the ED. Participants reported that patients generally express higher contentment when allowed to stay in their own homes. Paramedic participants shared that the home environment tends to foster increased receptiveness to engage in conversations and shared decision making.

*‘Patients are more relaxed in their own home, and they are more willing to listen to you….and discuss different pathways and more open to trying things to try and stay at home.’ – participant 12*

Participants also highlighted the quick reassurance a POC test could provide to patients, particularly if a decision not to convey the patient is made. Patient and public participants discussed the challenges of supporting a family member in the ED as opposed to in their own homes.

*‘Although it might seem the most painful and hard work [looking after a patient at home]. It actually probably won’t be, because when somebody is in hospital and you are waiting like 10 days for a test, it’s absolutely exhausting going in and out and doing all the other stuff.’ – participant 19*

### Navigating implementation challenges: balancing barriers and facilitators for successful implementation

Participants discussed numerous impediments and enablers associated with the implementation of POC tests in EMS, with a degree of overlap between factors serving as both facilitators and barriers.

***Logistics***

When discussing barriers and facilitators, logistics was frequently discussed. Participants emphasised several factors relating to POC tests in EMS, including user-friendliness, test consistency and replicability across different environments, storage, robustness, self-calibration to prevent burdening staff, compact size, power source accessibility, simple interpretability, minimal invasiveness, and rapid result generation. There was also support for a POC testing device that could offer multiple different tests.

*‘They’ve got to be really easy to use in a challenging environment.’ – participant 3*

***Organisational barriers and risks***

The out-of-hospital setting generally leans towards risk aversion due to the seriousness of the consequences, and this aspect was identified as a hindrance to implementation due to the limited potential for altering this established culture. Lack of support from managers with regards to decision making when using a POC test was highlighted.

Ambiguous results could potentially lead to unsafe patient discharges at the scene, particularly when non-diagnostic test outcomes are based on numerical scales or if they are detecting a biomarker that may change significantly within a short period of time, such as troponin. It was highlighted that paramedics should be educated when interpreting results to support their decision on conveyance.

The management of change was also anticipated to be a barrier during the implementation process. Different healthcare trusts currently face distinct challenges, such as escalating population numbers, regions lacking additional facilities, and a shortage of GPs (General Practitioners).

*‘Those barriers of lack of managerial support and risk aversion definitely need overcoming before everyone is going to be happy.’ – participant 1*

***Financial impact***

The introduction of POC tests could potentially have financial implications for ambulance services, including the cost of purchasing tests, component costs, and paramedic training. These changes might have advantages for providers beyond the ambulance service, but such benefits would need to be passed back to the ambulance service to incentivise and support adoption. Apprehension was voiced regarding the risk of duplicate testing if patients are transported to hospital, thereby increasing overall costs. Additionally, there is a prospect of resource allocation impact due to the time invested in calibrating the POC tests and ambulance staff dedicating more time to patient interactions when ED conveyance does not occur.

*‘The cost of the test and the machine and the supplies and the maintenance are all barriers.’ – participant 14*

***Coordination across the pathway***

Coordination across the patient pathway was identified as an important facilitator in the implementation of POC tests, and provides a basis for improved communication between healthcare professionals. Being able to have clear communication with clinicians ahead of admission is important to enable preparation and any required infection control. It would also support the triage and streaming of patients within the ED. GPs reported that having results from POC tests would be useful. The sharing of records would support coordination across the pathway more generally, but particularly when considering POC tests.

*‘You know the ambulance service is going to do something and the support has to be there from the other services to make it work.’ – participant 21*

***Clear purpose***

Establishing well-defined objectives for POC tests was considered integral to streamlining their implementation and potentially assisting ambulance staff in overcoming associated challenges. Their purpose must be clear, and paramedics must be able to interpret results effectively, using results to support decision making. Identifying key decision points influenced by a POC test would be a facilitator. Participants emphasised that POC testing serves as supplementary information to construct a broader clinical picture and evidence, without replacing clinical judgment. It was highlighted that patient overview could still be limited with just the POC test results. Various factors, including co-morbidities, individual patient characteristics, observations and other potential reasoning must be considered.

*‘You can spend hours doing lots of tests, but you have to be clear what you are doing with them.’ – participant 9*
*‘What we have got to be careful as an ambulance trust I think, is that the point of care testing is an addition. My anxiety with it is that perhaps some paramedics would use it as a rule out test.’ – participant 25*

### Building the foundations: key considerations for future research

Participants were aware of the study's aims and were subsequently prompted to reflect on the potential for future research. They emphasised the necessity for careful management of POC test implementation, highlighting the criticality of feasibility testing, particularly to ascertain the willingness of paramedics to use these tests. Participants stressed the importance of having a local study contact to facilitate study delivery; a lesson drawn from prior studies.

*‘It doesn’t matter what you have got if people can’t use them accurately and effectively.’ – participant 5*

The significance and practical aspects of training were also addressed. Participants stressed the need for comprehensive training to instil confidence in users and ensure appropriate interpretation of results to ensure a meaningful contribution to decision-making. While the importance of training was acknowledged, preferences regarding its delivery method varied, with advantages and drawbacks of both face-to-face and online training being discussed. Additional support, for example from a senior clinician who could be consulted by telephone, would also help paramedics to feel confident in their decision-making.

Regarding the selection of appropriate tests, participants emphasised the importance of choosing tests that would provide substantial benefits to many patients and support non-conveyance decisions. Participants noted the rapid pace of commercial developments and advocated for a flexible trial design capable of accommodating alternative tests.

Additional considerations included ensuring the availability of evidence supporting an analysis of cost-effectiveness, implementing effective quality control measures, engaging technicians, incorporating the patient voice in dissemination, addressing broader pathways alongside POC test implementation, and regularly capturing the opinions of paramedics.

*‘I think it is really important to talk to them [paramedics] and find out if this will make their daily work easier or not. Because that is the, if it doesn’t make their [paramedics] daily life easier, they will probably not use it.’ – participant 7*

## Discussion

Our findings indicate that the integration of additional POC testing could have positive impacts on patient care and system efficiency, but successful implementation requires the navigation of various challenges and considerations. Limited literature exists exploring perspectives on point-of-care (POC) testing in ambulance settings, particularly using qualitative approaches. Nonetheless, our findings broadly resonate with previous research. For example, a prior survey suggested ambulance-based POC testing was feasible in concept, but there were major barriers that needed to be addressed, and it would only be used if paramedics felt it would add value to early clinical care decisions. This aligns closely with our own observations. However, this survey focused on SARS-CoV-2 testing, and the added value of this specific test was questioned.

A key finding was the recognition among participants of POC testing's potential to alter patient pathways. One UK EMS provider organisation introduced POC testing and completed a service evaluation observing a 31% alteration in conveyance decisions, resulting in high levels of patient satisfaction and acceptance from paramedics as a decision-making tool ^4^. This small-scale, yet complex, project suggests that further exploration of the implementation of POC testing and paramedic clinical decision-making is warranted. Notably, the project did not include a cost-benefit analysis. Our findings suggest that the decision not to convey a patient may reduce stress or inconvenience for patients and their family. Non-conveyance is generally accepted by patients, but this also depends on the interpersonal skills of the healthcare professionals involved, who should ensure appropriate time is spent providing thorough information about the decision and clear guidance on what to expect if a follow-up is required ( 4, 13; www.eflm.eu/Hot-Topic-in-LM-POCT.pdf). 

Alongside the potential to alter pathways, participants also highlighted the potential impact on patient outcomes, emphasising the role of POC testing in expediting the decision-making process for specific treatments. Previous works suggests that administering antibiotics in the prehospital setting leads to decreased mortality rates, and lactate has also been used as a decision support tool for major trauma patients, by predicting the need for in-hospital transfusions of blood ^14^.

In other settings where POC testing is utilised, the benefits have been demonstrated through previous research. EDs currently rely on swift identification of critical conditions through laboratory testing, which guides 60-70% of clinical decisions ^15^. The use of POC testing in suitable situations within the ED has been proposed as an effective means to decrease time to treatment and enhance patient outcomes ^16^. POC testing has also been recommended for expediting discharge decisions in ambulatory care ^17^.

Some of the barriers highlighted by stakeholders have been reflected on previously, specifically the logistics of size, robustness, portability, usability, environment, and trained paramedics who can interpret the results accurately ( 13; www.eflm.eu/Hot-Topic-in-LM-POCT.pdf) and continued competency of staff are ongoing challenges, and preanalytical errors, including incorrect collection procedures, account for approximately 60-70% of errors in the laboratory setting. Therefore, training should be carefully considered in the design of any future trial ^4, 18^. Aligning with our findings, these factors need careful consideration when selecting individual POC tests as they may significantly influence paramedic use.

The findings from this study have highlighted the perceived key benefits and impact that POC testing could potentially bring to EMS, aligning with a stated aim of the NHS to reduce ED conveyance by either safely leaving patients at home or seeking treatment elsewhere ( www.england.nhs.uk/National-guidance-for-ambulance-clinician-referral-to-same-day-emergency-care.pdf; www.england.nhs.uk/planning-to-safetly-reduce-avoidable-conveyance-v4.0.pdf). These changes in patient pathway could consequently have a positive impact on fleet availability, enhancing the response capacity for patients in the community, and overall patient outcomes. Our study suggests that while the implementation of POC testing has perceived potential benefits, numerous considerations must be thoroughly evaluated, including alterations to the patient pathway, costs, and patient outcomes. The barriers identified, in keeping with previous research, present obstacles that should be carefully considered when designing future research relating to specific tests.

## Strengths and limitations

While the recruitment target was surpassed and participants provided rich, in-depth insights, the reliance on convenience and snowballing sampling methods may pose a limitation on generalisability, since this study primarily attracted individuals with an interest in POC testing. From a geographical standpoint, the participants were drawn from diverse regions and various employers throughout the UK and included three international participants. While this diversity offered a broad overview of thoughts and experiences, discerning which aspects were specifically influenced by their respective regions or employers posed a challenge. The input from commissioners of EMS services was limited, potentially creating a gap in responses, and hindering a comprehensive understanding of the broader context. Finally, specific tests were not thoroughly explored, so while broader perceptions were captured, these would need to be further developed in relation to specific tests and use cases.

## Conclusions

In this study participants indicated that the incorporation of POC testing in UK EMS has strong potential to alter patient pathways, enhance system efficiency, aid paramedic decision-making and reduce stress for patients and their families. Nevertheless, it is imperative to address and consider additional factors such as logistics, risks, financial implications, and coordination across the healthcare system. It is recommended that future research should prioritise these aspects for further exploration when considering specific tests.

## Data Availability

Our qualitative data from the semi-structured interviews is not able to be effectively de-identified at the individual level to an acceptable standard due to the specific job roles, geographical regions and experiences included. Additionally, the study participants did not provide consent for their data to be shared publicly. The data that support the findings of this study could be requested by emailing the corresponding author ( jessica.coggins@uwe.ac.uk), subject to approval by the chief investigators. Open Science Framework: Prehospital Point of Care Testing to Enable Decision-making (PrePoCTED), https://doi.org/10.17605/OSF.IO/C2Z34 ^9^. This project contains the following supplementary files: Interview topic guides. Data are available under the terms of the Creative Commons Attribution 4.0 International license (CC-BY 4.0).

## Software availability

NVivo 14 is a proprietary software, free alternatives such as QualCoder ( https://qualcoder.wordpress.com/) could be used.
