# Supplementary material for: Benefits, barriers and facilitators for out-of-hospital point of care testing: a qualitative study
Source: NIHR Open Res. 2024 Jun 27;4:38. [Version 1] doi: 10.3310/nihropenres.13583.1 (PMC12409259; doi:10.3310/nihropenres.13583.1)
Supplement: Peer review history

# Reviewer report: Reviewer response for version 1

**DOI:** 10.3310/nihropenres.14744.r36614

**Published:** 2025-09-03

**Authors:** Sarah Mills

**Affiliations:** 1 University of St Andrews, St Andrews, Scotland, UK

This manuscript presents a well-conceived and timely qualitative study exploring the implementation of Point of Care (POC) testing in UK Emergency Medical Services (EMS). It makes a valuable and original contribution to a relatively small but important field, particularly in the context of increasing system pressures and the need for innovation in urgent and emergency care. The study is methodologically sound, ethically robust, and clearly aligned with current NHS policy priorities. The inclusion of diverse stakeholders, including patient and public contributors, enhances the relevance and applicability of the findings. The thematic analysis is well-executed and yields insights with significant potential to inform future service design, commissioning, and clinical practice.

 To strengthen the manuscript further, several areas should be addressed. First, the presentation of Tables 2 and 3 requires attention, as both appear incomplete or inconsistently formatted. These tables should be fully presented with clear headings and explanatory captions. Second, the manuscript would benefit from a brief statement confirming whether data saturation was achieved, as this is a key consideration in qualitative research. Third, the coding process should be clarified, particularly how discrepancies between coders were resolved. Additionally, while the manuscript refers to a diverse stakeholder group, it would be helpful to include a summary of participant demographics, such as professional roles and geographical spread, to contextualize the range of perspectives.

 The findings are rich and policy-relevant, but the practical implications could be more explicitly articulated. For example, the discussion could be expanded to show how ambulance trusts or commissioners might use this evidence to guide procurement, training, or pathway redesign. Similarly, the section on future research would benefit from more specific suggestions, such as feasibility studies or economic evaluations, and the discussion of financial and organizational barriers could be enhanced by considering how clinical governance frameworks or decision support tools might mitigate risks associated with ambiguous test results and cultural resistance.

 Overall, this is a strong and important manuscript that deserves indexing. With minor revisions to improve clarity, completeness, and practical framing, it will make a meaningful contribution to the evidence base for EMS innovation and healthcare system improvement.

Is the work clearly and accurately presented and does it cite the current literature?

Yes

If applicable, is the statistical analysis and its interpretation appropriate?

Yes

Are all the source data underlying the results available to ensure full reproducibility?

No

Is the study design appropriate and is the work technically sound?

Yes

Are the conclusions drawn adequately supported by the results?

Yes

Are sufficient details of methods and analysis provided to allow replication by others?

Yes

Reviewer Expertise:

Urgent and unscheduled care, point of care testing.

I confirm that I have read this submission and believe that I have an appropriate level of expertise to confirm that it is of an acceptable scientific standard.

# Reviewer report: Reviewer response for version 1

**DOI:** 10.3310/nihropenres.14744.r36609

**Published:** 2025-08-29

**Authors:** Julie Shaw

**Affiliations:** 1 The Ottawa Hospital and EORLA, Ottawa, Ontario, Canada

Here, the authors present findings from structured interviews with experts in the field of POCT in EMS in England.  The authors touch upon barriers and potential benefits of POCT in EMS. The barriers and challenges make sense and highlight the need for clear guidelines/recommendations for any POCT performed outside hospital settings. My challenge with this study is that the findings lack detail. The questions asked in the interviews seem rather generic and high-level. What would be more useful to the field would be details. What specific POCT would be useful in EMS? How would these programs best be managed? What clinical pathways could POCT aid with? What decision supports are required? I feel these details are missing. I note that questions about specifics were included in the interview guide. Perhaps these answers were not obtained? If the authors are able to include more details and specifics, it would strengthen the paper.

Is the work clearly and accurately presented and does it cite the current literature?

Yes

If applicable, is the statistical analysis and its interpretation appropriate?

Not applicable

Are all the source data underlying the results available to ensure full reproducibility?

No source data required

Is the study design appropriate and is the work technically sound?

Yes

Are the conclusions drawn adequately supported by the results?

Partly

Are sufficient details of methods and analysis provided to allow replication by others?

No

Reviewer Expertise:

POCT and quality assurance

I confirm that I have read this submission and believe that I have an appropriate level of expertise to confirm that it is of an acceptable scientific standard, however I have significant reservations, as outlined above.

# Reviewer report: Reviewer response for version 1

**DOI:** 10.3310/nihropenres.14744.r32991

**Published:** 2024-10-31

**Authors:** Markus Skrifvars

**Affiliations:** 1 University of Helsinki, Helsinki, Finland

Markus Skrifvars, University of Helsinki, Finland

 Regarding the paper “Benefits, barriers and facilitators for out-of-hospital point of care testing: a qualitative study”. This study uses mainly interviews with different stakeholders aiming at getting their views on the use of “point of care” testing the EMS setting.

 I have the following comments:

- It may not be clear to all readers what is actually mean by “point-of-care” testing. I guess here the authors refer to point-of-care laboratory tests that can be done by the EMS personnel on site. However, this is a wide definition. Do the authors mean mainly blood gas (venous or arterial) testing including lactate? How about glucose, with simple glucose monitors? Or are the authors referring to CPR, troponin, BNP etc? A Table with some various tests would be useful for the reader.
- In the discussion the authors mention the measurement of lactate in sepsis and trauma as a mean to decide on further treatment. This is one aspect but on the other hand if the patient has such a major trauma that in-hospital blood transfusion is possible one can really discuss whether anything else than assessment and treatment of the ABCs are really needed. Any test that takes time will increase the time to hospital admission. And in the hospital multiple things happen at the same time, including proper assessment of laboratory tests etc. 
- Continuing on from the above on key problem with POC testing for example in suspected sepsis is that there will be a group of patients that are so sick that that they require rapid transport regardless of any POC test. Then there is a group that are so well that regardless of the POC test the patient can probably go to the ED with their own transport. The POC tests possible real effect is likely to occur in the middle group, where the situation is unclear. This is a hallmark of the use of any testing in medicine, take into account the pretest probability! What has happened in medicine in the hospital is that we take a lot of tests “just in case” as some form of “screening”. I doubt that this is effective. It is likely that the same will happen with POC testing in the pre-hospital setting. How can we avoid this? Is this something that the authors could discuss a bit more?
- How about the use of patient vital signs as a mean to trigger POC testing? Again if the vital signs are deranged enough for example with high NEWS scores it is quite possible that POC testing will not make too much of a difference.
- Blood glucose testing in the pre-hospital setting as a mean to manage diabetic patients is standard of care. Is this also POC testing?
- A big problem for example with sepsis screening with biomarkers is the slow rise in the hyperacute setting. With a very severe sepsis the CRP may be low but the after a day or two it will increase. So the use of CPR for screening for severe infection may not work as “screen out” test. If the level is low one cannot be sure that the patient does not have sepsis. But if the CRP is high it increases the likelihood of sepsis if there are no other explanations (major trauma, inflammatory disease etc.)

All in with some modification this will study will interest readers.

Is the work clearly and accurately presented and does it cite the current literature?

Partly

If applicable, is the statistical analysis and its interpretation appropriate?

Not applicable

Are all the source data underlying the results available to ensure full reproducibility?

Partly

Is the study design appropriate and is the work technically sound?

Partly

Are the conclusions drawn adequately supported by the results?

Partly

Are sufficient details of methods and analysis provided to allow replication by others?

Partly

Reviewer Expertise:

Emergency care, intensive care

I confirm that I have read this submission and believe that I have an appropriate level of expertise to confirm that it is of an acceptable scientific standard, however I have significant reservations, as outlined above.
